# Anthocyanin Metabolites in Human Urine after the Intake of New Functional Beverages

**DOI:** 10.3390/molecules25020371

**Published:** 2020-01-16

**Authors:** Vicente Agulló, Débora Villaño, Cristina García-Viguera, Raúl Domínguez-Perles

**Affiliations:** 1 Phytochemistry and Healthy Foods Lab. Group of Quality, Safety and Bioactivity of Plant Foods. Department of Food Science and Technology, (CEBAS-CSIC), University Campus Espinardo 25, 30100 Murcia, Spain; 2 Universidad Católica San Antonio de Murcia (UCAM), Department of Pharmacy, Faculty of Health Sciences, Campus de los Jerónimos, Guadalupe, 30107 Murcia, Spain

**Keywords:** dietary intervention, maqui, juice, anthocyanins, bioavailability, UHPLC-ESI-QqQ-MS/MS

## Abstract

Sugar intake abuse is directly related with the increase of metabolic diseases such as type 2 diabetes, obesity, and insulin resistance. Along this line, the development of new beverages using alternative sweeteners could help with combatting the pathophysiological disorders associated to the consumption of sugar. To provide evidence on this issue, in the present work, the bioavailability of anthocyanins was evaluated after the acute ingestion of a new maqui-citrus-based functional beverage rich in polyphenols, and supplemented with a range of sweeteners including sucrose (natural high caloric), stevia (natural non-caloric), and sucralose (artificial non-caloric), as an approach that would allow reducing the intake of sugars while providing bioactive phenolic compounds (anthocyanins). This approach allowed the evaluation of the maximum absorption and the diversity of metabolites excreted through urine. The beverages created were ingested by volunteers (*n* = 20) and the resulting anthocyanin metabolites in their urine were analyzed by UHPLC-ESI-MS/MS. A total of 29 degradation metabolites were detected: Caffeic acid, catechol, 3,4-dihidroxifenilacetic acid, hippuric acid, *trans*-ferulic acid, 2,4,6-trihydroxybenzaldehyde, *trans*-isoferulic acid, and vanillic acid derivatives, where peak concentrations were attained at 3.5 h after beverage intake. Sucralose was the sweetener that provided a higher bioavailability for most compounds, followed by stevia. Sucrose did not provide a remarkably higher bioavailability of any compounds in comparison with sucralose or stevia. The results propose two sweetener alternatives (sucralose and stevia) to sucrose, an overused high calorie sweetener that promotes some metabolic diseases.

## 1. Introduction

The abuse of sugar intake in the population is based on the stimulation provided by the sweetness provided by compounds such as sucrose, glucose, and saccharine liquid, through the stimulation of the shared brain reward pathways [1,2,3,4]. This has boosted the consumption of sugar-sweetened beverages, critically contributing to the amount of unhealthy sugar consumed through the human diet [5]. Indeed, the raising consumption of sweeteners occurring during the last decades has been associated to the growing prevalence of metabolic diseases, namely type 2 diabetes, as a result of their contribution to obesity and insulin resistance [6]. In this regard, Bernstein et al., 2012 reported that sugar-sweetened beverages may be related with the increased risk of stroke in humans, as evidenced by epidemiological studies [7].

In order to prevent the deleterious effect of sugar intake in modern societies, global strategies have been focused on lifestyle, suggesting the need to change dietary habits, in parallel to the increase of physical exercise [8,9]. With this objective, a number of recent studies have discussed the interest of including fruits in the human diet, as sources of bioactive phytochemicals, more specifically anthocyanins. Along this line, this strategy is supported by the cumulative data available on the biological effects of these compounds on glycolipid metabolism, reducing fasting and 2 h postprandial glucose, and modulating the level of glycemic biomarkers and low density lipoprotein (LDL) [10,11]. However, the bioavailability of the bioactive polyphenols reported in the literature is low, being estimated to lie in a range between 5% and 10% of the total intake, after intestinal absorption [12]. Despite this limited bioavailability, which could indicate a poor biological relevance regarding the control of glucose metabolism, a recent study has suggested a positive interaction between sucrose and phenolic compounds that could inhibit the formation of insoluble protein-proanthocyanidin aggregates, increasing their bioaccessibility and bioavailability [13]. On the other hand, an interest has been observed of using stevia, a *trans*-glycosylated food additive, to improve the solubility and bioavailability of phenolic compounds [14]. This is in agreement with the recommendation by the World Health Organization (WHO) for reducing the intake of free sugars to less than 10% of the total energy intake in adults and children, thus tackling the diverse pathophysiological situations associated to its consumption [15]].

Fruits selected for the development of new beverages feature a high content of bioavailable and bioactive compounds, and contribute to lowering the risk of certain diseases (including cardiovascular diseases) [16,17,18]. Hence, due to the importance of fruits rich in bioactive compounds, maqui (*Aristotelia chilensis* (Mol.) Stuntz), a purple blackberry from Chile and Argentina, has been characterized for its phytochemical composition and biological potential [19,20], resulting in its promotion as a natural source of bioactive compounds (mainly flavonoids, especially anthocyanins, and, in a lesser extent, flavonols, catechins, proanthocyanidins, ellagitannins and phenolic acids), featured by a high antioxidant capacity, cardioprotection properties, and inhibition of adipogenesis, inflammation, and diabetes symptoms [16].

According to these antecedents, the present work studies the influence of sweeteners on the bioavailability of anthocyanins on healthy humans after an acute administration of a polyphenol-rich maqui berry/citrus beverage, supplemented with a range of sweeteners including sucrose (natural high caloric), stevia (natural non-caloric), and sucralose (artificial non-caloric). These sweeteners were selected accordingly to establish a comparison between a classical natural and high caloric sweetener (sucrose) and two non-caloric alternatives. Stevia was selected as a natural, emergent sweetener and sucralose as an artificial and widely-used sweetener. In order to fulfill this objective, urinary metabolites from maqui berry anthocyanins were searched for based on previous research studies [21,22].

## 2. Results and Discussion

### 2.1. Anthocyanin Content of Juices

In order to determine the bioavailability of the anthocyanins present in the maqui-citrus beverages created using three separate sweeteners (stevia, sucralose, and sucrose), the anthocyanin composition of the juices was characterized. Thus, the presence of 8 anthocyanins was observed, with the most abundant being Dp 3-*O*-sambubioside, Dp 3-glucoside, Dp 3,5-*O*-diglucoside, and Dp 3-*O*-glucoside, with average concentrations of 3.15, 3.49, and 2.93 mg/100 mL, on average, and respectively. The second most-abundant group of compounds according to their concentration in the juices was the co-eluting Cy 3-*O*-sambubioside-5-*O*-glucoside and Cy 3,5-*O*-diglucoside (1.48 mg/100 mL, on average) and Dp 3-*O*-sambubioside (1.10 mg/100 mL, on average). The lowest concentration corresponded to Cy 3-*O*-sambubioside (0.40 mg/100 mL, on average) and Cy 3-*O*-glucoside (0.55 mg/100 mL, on average). No significant differences between the anthocyanin content of the array of maqui-citrus beverages was observed neither when considering individual nor total anthocyanins (Table 1).

### 2.2. Qualitative Analysis of Urine Metabolites of Maqui-Citrus Juice Anthocyanins

The characterization of the diversity of maqui anthocyanin metabolites excreted was done in 24-h urine after the ingestion of 330 mL of maqui-citrus juice by healthy volunteers with the aim of evaluating possible differences due to the sweetener employed (stevia, sucralose, and sucrose). The analysis of the different urines evidenced the occurrence of 29 diverse phenolic metabolites derived from the list of anthocyanin metabolites monitored, referred to in Table 2. More specifically, the compounds identified were Caffeic acid (CA), CA-glucuronide, CA-sulfate, CA-glucuronide-sulfate, Catechol (CAT)-di-glucuronide, CAT-sulfate, CAT-glucuronide-sulfate, 3,4-Dihidroxifenilacetic acid (DHPAA), DHPAA-glucuronide, DHPAA-di-glucuronide, DHPAA-sulfate, DHPAA-glucuronide-sulfate, DHPAA-di-sulfate, Hippuric acid (HA), HA-glucuronide, HA-di-glucuronide, HA-sulfate, *Trans.*-Ferulic acid (TFA)-glucuronide, TFA-di-glucuronide, TFA-sulfate, TFA-di-sulfate, 2,4,6-Trihidrobenzaldehid (THBA)-glucuronide, THBA-sulfate, TIFA-sulfate, Vanillic acid (VA), VA-glucuronide, VA-sulfate, VA-glucuronide-sulfate and VA-di-sulfate. Interestingly, the precursor anthocyanins (Dp and Cy) were not detected. This finding could be attributed to the degradation during digestion with the following forming of glucurono-, sulfo-, or methyl-derivatives in the proximal gastrointestinal tract, although a mechanism for the absorption of anthocyanin glycosides is still highly speculative [22].

Some anthocyanin metabolites referred to above were found in the urine samples of a reduced number of volunteers; for instance, CA-glucuronide-sulfate, CAT-di-glucuronide, CAT-glucuronide-sulfate, DHPAA-di-glucuronide, DHPAA-glucuronide-sulfate, DHPAA-di-sulfate, HA-di-glucuronide, HA-glucuronide, TFA-di-glucuronide, TFA-di-sulfate, and VA-di-sulfate. These compounds were present in quantifiable amounts, but the limited number of volunteers who excreted these molecules means that the results were not representative of the general population. The occurrence of these metabolites may be due to the inter-individual variations of metabolic traits [23]. which are also responsible for the dispersion of the metabolite concentration in the present work. On the other hand, CA, CA-glucuronide, CA-sulfate, CAT-sulfate, 3,4-DHPAA, DHPAA-glucuronide, DHPAA-sulfate, HA, HA-sulfate, TFA-glucuronide, TFA-sulfate, THBA-glucuronide, THBA-sulfate, TIFA-sulfate, VA, VA-glucuronide, VA-glucuronide-sulfate, and VA-sulfate were identified and quantified in the urine sample from of all the volunteers.

### 2.3. Assessment of the Concentration of Anthocyanins Metabolites in Urine Samples

**Figure 1:**
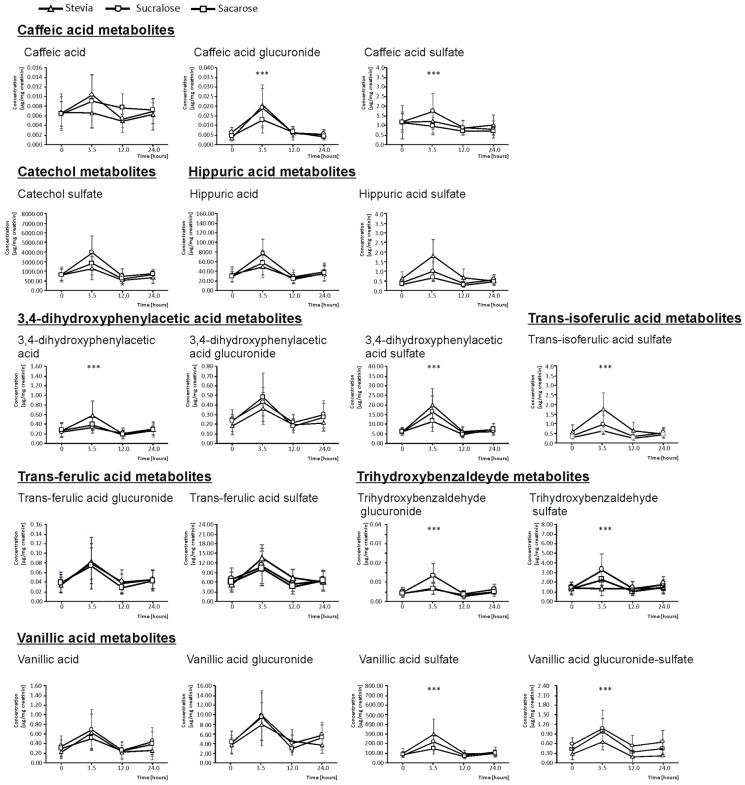
Content (mean ± SD, *n = 20*) of single anthocyanins metabolites (caffeic acid, caffeic acid-glucuronide, caffeic acid-sulfate, catechol-sulfate, 3,4-dihydroxyphenylacetic acid, 3,4-dihydroxyphenylacetic acid-glucuronide, 3,4-dihydroxyphenylacetic acid-sulfate, hippuric acid, hippuric acid-sulfate, *trans*-ferulic acid-glucuronide, *trans*-ferulic acid-sulfate, 3,4-dihydroxyphenylacetic acid -glucuronide, 3,4-dihydroxyphenylacetic acid-sulfate, *trans*-isoferulic acid-sulfate, vanillic acid, vanillic acid-glucuronide, vanillic acid-glucuronide-sulfate, and vanillic acid-sulfate) in basal urine and 3.5,12, and 24-h urine of healthy volunteers after ingesting 330 mL of maqui-citrus juices developed using as sweeteners stevia (∆), sucralose (O), and sucrose (□). Significantly different bioavailabilities according to an analysis of variance (ANOVA) and duncan’s multiple rank test were found at *p* < 0.001 (***).

In urine, the quantification of the anthocyanin metabolites excreted was performed on basal urine (0 h), as well as on urine excreted between 0 and 3.5 h, 3.5–12 h, and 12–24 h. The excretion kinetics for all the compounds matched between the volunteers ingesting the three types of juices evaluated, showing the highest concentration for all compounds identified at 3.5 h after the consumption of the beverages (Figure 1). For this reason, all the concentrations described and analyzed statistically below refer to the timeframe 0–3.5 h after the ingestion.

As mentioned before, no precursor anthocyanins (Dp or Cy) were found in urine. However, an array of metabolites was detected and quantified, being the focus of the bioavailability for these compounds. For example, CA, a common degradation product of anthocyanins that is formed in the small intestine as a result of a pH-mediated breakdown of anthocyanins [24], was identified. Indeed, this phase I reaction could be responsible, to a high extent, for the limited bioavailability of the precursor anthocyanin. The total urine concentration of CA (expressed as the sum of CA and its phase II esterified metabolites) was 4.75 µg/mg creatinine for sucralose, the sweetener that provided the highest bioavailability which surpassed the excretion recorded, when ingesting the beverages with stevia and sucrose added, by 6.7% and 22.4%, respectively. When analyzing the effect of the sweetener regarding the individual metabolites (unesterified CA, CA-glucuronide, and CA-sulfate), in all the cases the highest value corresponded to sucralose (0.03, 0.04, and 4.68 µg/mg creatinine, respectively), relative to the bioavailability when using stevia (a 16.0% lower bioavailability regarding CA), sucrose (a 24.7% lower bioavailability of caffeic acid-glucuronide), and both sucrose and stevia (a 14.6% lower bioavailability, on average, for CA-sulfate). These differences were statistically significant for CA-glucuronide (*p* < 0.001) and CA-sulfate (*p* < 0.01) (Figure 1).

In turn, CA is metabolized to TFA and TIFA via enterocyte-based methylation and sulfation/glucuronidation steps [22]. The urine concentration of TFA, expressed as the sum of the concentrations of the individual metabolites (unesterified and phase II metabolites), was 30.00 µg/mg creatinine, on average, for all beverages, with no significant differences found between them. Alternatively, TIFA-sulfate was the only metabolite of TIFA found in urine, and its concentration was 91.16 µg/mg creatinine in the urine of the volunteers who ingested the sucralose-sweetened beverage (which had the highest bioavailability). As for the beverages with stevia and sucrose, the TIFA concentration achieved was 28.7% (significantly) lower, on average. However, further studies are needed to learn more about the influence of sweeteners in the different metabolic pathways, since, to the best of our knowledge, no research on this issue has provided evidence suggesting the modulation of the diverse metabolic pathways as a consequence of the diverse sweeteners.

An additional metabolite of anthocyanins, THBA, was found in urine after the intake of maqui-citrus juices. This metabolite is synthesized as a result of the anthocyanin degradation mediated by aldehyde [25]. Again, its highest total urine concentration, as the sum of THBA-glucuronide and THBA-sulfate, corresponded to the intake of sucralose-sweetened juices (7.71 µg/mg creatinine), while when ingesting juices created using sucrose and stevia, 23.5% and 29.9% lower concentrations were obtained. When analyzing the effect of the sweetener in regard to the bioavailability of individual metabolites (glucuronide and sulfate), in both cases the highest value corresponded to sucralose (0.03 and 7.68 µg/mg creatinine, respectively), which significantly surpassed the urine concentration provided by stevia and sucrose, are related to THBA-glucuronide (by 34.9%, on average; *p* < 0.001) and relative to THBA-sulfate (by 29.8% and 23.4%, respectively; *p* < 0.001) (Figure 1).

Another CA degradation pathway gives rise to 3, 4-dihydroxyphenil propionic acid, an intermediate metabolite that ultimately provides DHPAA. This reaction is developed with the participation of mammalian enzymes and colonic microbiota [22] and, according to the results obtained in the present work, it is significantly affected by the separate sweeteners. Hence, the highest total concentration of DHPAA in urine corresponded to volunteers who ingested maqui-citrus juices sweetened with stevia (41.35 µg/mg creatinine), while the intake of beverages created using sucralose and sucrose provided urine concentrations which were 12.2% and 23.4% lower relative to stevia. The analysis of the relative contribution of the diverse DHPAA metabolites found (unesterified DHPAA and DHPAA-sulfate), showed that the highest value also corresponded to stevia (1.35 and 39.06 µg/mg creatinine, respectively), which significantly surpassed the bioavailability provided by sucralose and sucrose, in regard to DHPAA (by 17.3%, on average; *p* < 0.001) and the bioavailability provided by sucrose, concerning DHPAA-sulfate (by 24.7%; *p* < 0.001) (Figure 1).

Lastly, the metabolic pathway of anthocyanins transforms DHPAA to CAT, HA, and VA, as well as their phase II conjugates [21,22]. With respect to CAT, the metabolite detected was CAT-sulfate, and its urinary excretion did not show significant differences between sweeteners although the highest values corresponded to sucralose (8934.8 µg/mg creatinine), which showed 22.3% higher values than stevia and sucrose, on average. The urine excretion of HA and HA-sulfate gives rise to the highest concentrations for sucralose (180.02 µg/mg). Regarding these anthocyanin metabolites, stevia and sucrose showed significantly lower amounts (16.9%, on average; *p* < 0.001) than sucralose. This value was provided almost entirely by HA. Focusing on HA-sulfate, the excretion value was 3.20 µg/mg creatinine for stevia. The remaining sweeteners showed a 41.9% lower excretion, on average. In the case of VA, the total urine concentration calculated as the sum of its unesterified metabolites and their phase II conjugates was 595.98 µg/mg creatinine for stevia. However, when ingesting the juices created using sucralose and sucrose, 16.3% and 33.2% lower amounts were excreted. In this case, this value was provided almost entirely by VA-sulfate. Focusing on VA and VA-glucuronide excretion rates, no significant differences were found between sweeteners. However, the VA-glucuronide-sulfate excretion value was 3.46 µg/mg creatinine for sucralose, which significantly surpassed the bioavailability provided by stevia (a 52.4% lower; *p* < 0.001) (Figure 1).

The assessment of the urinary excretion of CAT, HA, and VA indicated much higher concentrations relative to the level of their precursors in the juices ingested. This prompted us to hypothesize that the presence of CAT, HA, and VA in urine could be the result of other dietary and endogenous sources, as demonstrated for HA [24]. This could contribute to the detection of very high urine concentrations that could not be otherwise explained by the single ingestion of the anthocyanins provided by maqui to the beverages.

In addition, although the Dp class of anthocyanins was the most-commonly found in the drinks, representing up to 81.0% of the total anthocyanins, on average, for all beverages, its direct metabolite (GA), a fragmentation product of delphinidin glycosides, was not detected [26,27]. This could be the result of the degradation of GA, the most relevant DP metabolite in the human body, to form different molecules such as 4-*O*-methylgallic acid, pyrogallol, 2-*O*-methylpyrogallol, and resorcinol [28]. With respect to the relative contribution of the phase II metabolites of the compounds monitored, from the results retrieved, it was observed that sulfated compounds were the most frequently found of the detected compounds, representing about 83.6% of the total excretion, on average.

Moreover, sucralose was the sweetener that provided the highest bioavailability for most compounds (CA, CAT, THBA, and TIFA derivatives, DHPAA-sulfate, HA and VA-glucuronide-sulfate), followed by stevia (DHPAA, HA-sulfate, and VA-sulfate). Sucrose did not provide a remarkably higher bioavailability of any compound as compared with sucralose or stevia, due to intestinal sugar carriers, which may play an important role in flavonoid absorption [29]. A study carried out by Mülleder et al. showed that the addition of sucrose to juices decreased the total excretion of anthocyanins [30].

The urinary metabolites profile, unesterified and their phase II conjugates, found in the volunteer samples after the intake of the maqui-citrus based beverage matched with the degradation metabolites of cyanidin after intake, previously reported by Kay et. al. [22].

### 2.4. Principal Component Analysis

**Figure 2:**
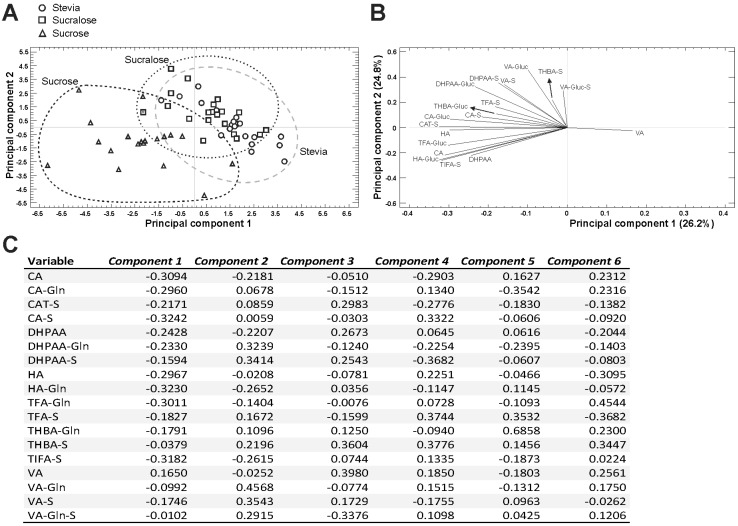
Principal component analysis (PCA) of the urine metabolites of anthocyanins ingested through newly developed citrus-maqui juices using different sweeteners (stevia, sucralose, and sucrose). Dot plot indicating the samples classification for the two first Principal Components Score plots (**A**), loading plot showing eigenvectors for the two principal components that account for at least 50.0% of the total variance (**B**), and Table indicating the components weights (**C**). CA, caffeic acid; CA-Gln, caffeic acid-glucuronide; CAT-S, catechol-sulfate; CA-S, caffeic acid-sulfate; DHPAA, 3,4-dihidroxifenilacetic acid; DHPAA-Gln, 3,4-dihidroxifenilacetic acid-glucuronide; DHPAA-S, 3,4-dihidroxifenilacetic acid-sulfate; HA, hippuric acid; HA-Gln, hippuric acid-glucuronide; TFA-Gln, *trans*-ferulic acid-glucuronide, TFA-S, *trans*-ferulic acid-sulfate, THBA-Gln, 2,4,6-trihidrobenzaldehid-glucuronide, THBA-S, 2,4,6-trihidrobenzaldehid-sulfate; TIFA-S, *trans*-isoferulic acid-sulfate; VA, vanillic acid; VA-Gln, vanillic acid-glucuronide, VA-S, vanillic acid-sulfate, VA-Gln-S, vanillic acid-glucuronide-sulfate.

After assessing the urinary metabolites profile detecting, the data were examined further using PCA, a statistical technique that simplifies a complex data set by reducing the number of variables (Figure 2). This (theoretical) classification contributed to the identification of sweeteners according to the urine metabolites profile of volunteers, thus indicating actual differences between them that will be much useful for the design of future nutritional trials. Hence, 18 components representing the total variance (100.0%) were extracted, with the first six explaining 68.2% of the variance. Of the total variance, 26.2% was explained by PC1, being mostly attributable to CA, CA-Glucuronide, CA-Sulfate, HA-Glucuronide, TFA-Glucuronide, and TIFA-Sulfate. With respect to PC2, the concentrations of DHPAA-Glucuronide, DHPAA-Sulfate, VA-Glucuronide, VA-Sulfate, and VA-Glucuronide-Sulfate were the most relevant contributors. The percentage of the variance explained by PC2 was 24.8%. Finally, PC 3, 4, 5, and 6 explained 7.0%, 4.4%, 3.8%, and 3.1% of variance, respectively.

The PCA developed using the concentrations of bioavailable metabolites of anthocyanins provided clusters alongside the PC1 vs PC2 plot (Figure 2A). Indeed, three clusters were clearly separated, with intermediate (positive and negative) scores for PC1 (stevia and sucralose, -clusters 1 and 2-), negative scores for PC1 (sucrose -cluster 3-). Regarding the ordinates axis (PC2), clusters 1–3 displayed intermediate scores that were more evident for clusters 1 and 2, whilst cluster 3 presented mainly negative values.

The analysis of the samples’ distribution, jointly with the eigenvectors informing on the weight of each variable and the correlation between them, indicated the relationship of the observed classification with the abundance of compounds in the samples under evaluation (Figure 2B). In this sense, the positive contribution of VA to PC1 suggests a close correlation between the samples distribution and clusters through the abscises axis, whilst the growing content of all additional individual metabolites contributed to negative values along this axis (Figure 2B).

## 3. Material and Methods

### 3.1. Chemicals and Reagents

The standards used for quantification purposes, caffeic (CA), gallic (GA), 3,4-dihidroxifenilacetic (DHPAA), hippuric (HA), *trans*-ferulic (TFA), *trans*-isoferulic (TIFA), vanillic acids (VA), 2,4,6-trihidroxibenzaldehid (THBA), and catechol (CAT), were obtained from Sigma Aldrich (St. Louis, MO, USA). Cyanidin (Cy) 3-*O*-glucoside and delphinidin (Dp) 3-*O*-glucoside were purchased from TransMIT (Geiben, Germany). Formic acid and acetonitrile were obtained from Fisher-Scientific (Loughborough, UK). All solutions were prepared with ultrapure water from a Milli-Q Advantage A10 ultrapure water purification system (Millipore, MA, USA).

### 3.2. Juice Preparation and Characterization of the Phenolic Content

Fresh, dry organic maqui berry powder was provided by Maqui New Life S.A. (Santiago de Chile, Chile). Cítricos de Murcia S.L. (Ceutí, Spain) and AMC Grupo Alimentación Fresco y Zumos S.A. (Espinardo, Spain) provided the citrus juices. Sucrose was provided by AB Azucarera Iberia S.L. (Madrid, Spain), Stevia by AgriStevia S.L. (Murcia, Spain), and Sucralose by Zukan (Murcia, Spain).

For preparing the maqui-citrus beverages, maqui powder was mixed with citrus juices to obtain the base beverage. Then, the three selected sweeteners were added, in different concentrations, in order to obtain an acceptable taste and to obtain the different beverages analyzed in the present work. The beverages underwent a pasteurization treatment through the application of 85 °C for 58 s. Afterwards, the mixtures were bottled and stored at 5 °C until being consumed by the volunteers.

As a preliminary task, the beverages developed were characterized on their polyphenolic composition. With this objective, the juices were centrifuged at 10,500 rpm, for 5 min (Sigma 1–13, B. Braun Biotech International, Osterode, Germany). The supernatants were filtered through a 0.45 µm PVDF filter (Millex HV13, Millipore, Bedford, MA, USA) and analyzed by RP-HPLC-DAD. The identification and quantification of anthocyanins was performed by applying the method previously reported [16,31]. Briefly, a chromatographic analysis of samples (10 µL), for the identification and quantification of anthocyanins was carried out on a Luna 5µm C18(2)100 Å column (250 × 4.6 mm), using Security Guard Cartridges PFD 4 × 3.0 mm, both supplied by Phenomenex (Torrance, CA, USA). The solvents used for the chromatographic separation were Milli-Q water/formic acid (95.0:5.0, *v*/*v*) (solvent A) and methanol (solvent B), in a linear gradient (time, %B) (0, 15%); (20, 30%); (30, 40%); (35, 60%); (40, 90%); (44, 90%); (45, 15%), and (50, 15%), using an Agilent Technologies 1220 Infinity Liquid Chromatograph, equipped with an autoinjector (G1313, Agilent Technologies) and a Diode Array Detector (1260, Agilent Technologies, Santa Clara, CA, USA). Chromatograms were recorded and processed on an Agilent ChemStation (Santa Clara, CA, USA) for LC 3D systems. The flow rate was 0.9 mL/min. The quantification of anthocyanins was done on UV chromatograms recorded at 520 nm as cyanidin-3-*O*-glucoside at 520 nm and expressed as mg per 100 mL of juice.

### 3.3. Experimental Design and Samples Collection

A double-blind, randomized, cross-over clinical study was conducted on overweight individuals (*n* = 20). The study and protocol were approved by the official Ethical Committee of Clinical Studies (CEIC) from the General University Hospital Morales Meseguer (Murcia, MU, Spain) (Murcia), as well as the Ethical Committee from the Catholic University of Murcia (UCAM) (Murcia, MU, Spain) and registered at ClinicalTrials.gov (NCT04016337). The inclusion criteria were: Individuals who were healthy, non-smoking, overweight (between 24.9 and 29.9 kg/m^2^ following WHO criteria), aged 35–55 years, non-dyslipidemic and normotense, with no chronic illnesses and not taking any medication.

**Figure 3:**
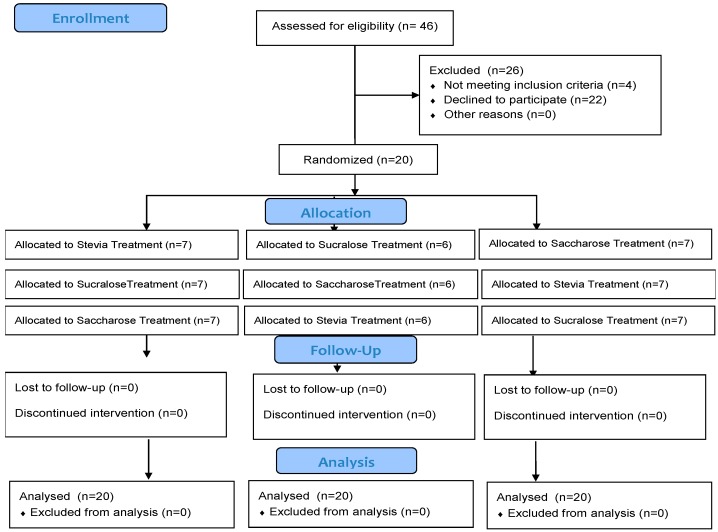
Consort flow diagram of the study.

After interviewing 46 individuals to assess their eligibility, 26 were not included in the study, as 4 did not fulfil the inclusion/exclusion criteria and 22 declined to participate. A total number of 20 volunteers were included in the study, as can be observed in Figure 3, according to the CONSORT 2010 statement [32]. There were no drop-outs. The basal characteristics of the volunteers are described as mean ± standard deviation (SD) for the following variables: Age (43 ± 8; years), Sex (16 men and 4 women), Weight (85.4 ± 10.4; kg), Height (1.77 ± 0.09; meters), BMI (27.25 ± 2.43; kg/m^2^) and Fat mass percentage (29.6 ± 8.1).

Volunteers were provided with written information about the study and all of them signed the informed consent form. All the participants had to follow a strict menu that was low in polyphenols and added sugars, created by a dietitian, for the 2 days before the intervention as well as the day of the intervention.

The day of the intervention, each volunteer received, on fasting conditions, one of the test drinks (330 mL), with one particular sweetener added (either stevia, sucralose or sucrose). Bottles were codified, thus the composition of each bottle was unknown to the volunteer, as well as to the researchers who provided the drinks and collected and processed the samples. The compliance was controlled as all volunteers were allocated to the same room for the intake of the drink, at the Catholic University of Murcia (UCAM).

After the intake of the test drink, volunteers were transferred to a clinical setting of the Nursing Department at the University, where they remained for the first 3.5 h after the intervention. Afterwards, urine containers were provided with instructions on collection of samples, volume measurement and storing. The day after, the volunteers provided the urine samples.

**Figure 4:**
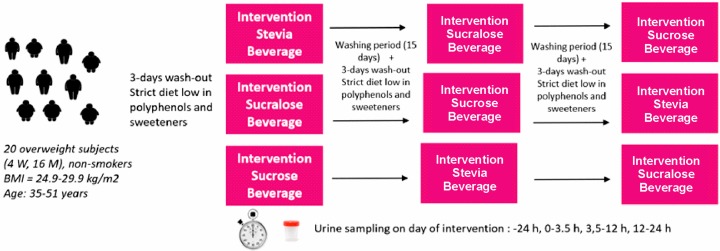
Cross-over study design.

Urine samples were collected 24 h earlier (0 point), as well as in the following intervals: 0–3.5 h, 3.5–12 h, 12–24 h. For each time-point, urine volumes were recorded and the samples were immediately aliquoted and stored at −80 °C until processing with the analytical procedures developed in the present work, according to the procedure described later in Section 3.4. (*Urine sample processing and analysis by UHPLC*-*ESI*-*MS/MS*). After 15 days, the process was repeated again, with each volunteer ingesting another drink, until all the drinks were consumed by all the volunteers (3 rounds). Figure 4 shows the cross-over study design. Analyses were performed once each period was finished and in the same batch to minimize analytical variations.

The total volume of each urine interval was recorded to calculate the absolute amounts of the compounds and metabolites excreted in the study period. Also, creatinine content was determined to normalize the concentrations of metabolites in urine as µg compound/mg creatinine, to control for differences in urine volumes.

The creatinine concentration in the urine samples was determined with the A-15 auto-analyzer (Biosystems^®^, Pleasenton, CA, USA). To verify the accuracy of the measurement procedure, the Biochemistry Control Urine and calibration of the device was performed, following our internal quality control scheme. The creatinine in the sample reacts with picrate in an alkaline medium, forming a colored complex (Jaffé method), that is monitored and quantified at 505 nm by the auto-analyzer. Concentrations are expressed as mg/dL [33].

### 3.4. Urine Samples Processing and Analysis by UHPLC-ESI-MS/MS

Urine samples (1 mL) were thawed and diluted 1:2 (*v*/*v*) in MilliQ-water/formic acid (99.9/0.1, *v*/*v*) and centrifuged at 15,000 *g* for 10 min, at 5 °C (Sigma 1–16, B. Braun Biotech International, Osterode, Germany). Afterwards, supernatants were filtered through 0.45 µm PVDF filters (Millex HV13, Millipore, Bedford, MA, USA) and stored at −20 °C until analysis by UHPLC-ESI-MS/MS.

The identification and quantification of anthocyanin metabolites was performed by applying the method previously reported by Ludwig et al. (2015) with some modifications [24]. The analysis of samples for the profile and concentration of anthocyanin metabolites was carried out on an Ascentis Express F5 column (5 cm × 2.1 mm; 2.7 µm) (Sigma, Osterode, Germany). The solvents used for the chromatographic separation were Milli-Q water/formic acid (99.9:0.1, *v*/*v*) (solvent A) and acetonitrile/formic acid (99.9:0.1, *v*/*v*) (solvent B), with a linear gradient (time, %B) (0, 10%); (1, 10%); (10, 60%); (11, 80%); (13, 80%); (13.01, 10%), and (14.50, 10%); using an UHPLC system coupled with a triple quadrupole tandem mass spectrometer model 6460 (Agilent Technologies, Waldbronn, Germany), operating in multiple reaction monitoring (MRM) and negative/positive electrospray ionization (ESI) modes. The volume injected and flow rate were 10 µL and 0.2 mL/min, respectively. The MS parameters at the optimized conditions were gas temperature 325 °C; gas flow 10 L/min; nebulizer 40 psi; sheath gas heater 275 °C; sheath gas flow 12; capillary voltage 4000–5000 V; Vcharging 1000–2000. Data acquisition and processing were performed by using MassHunter software version B.08.00 (Agilent Technologies, Walbronn, Germany). The diverse phenolic compounds in the samples were identified by comparing them with authentic, analytical-grade standard compounds.

### 3.5. Statistical Analysis

Quantitative data are presented as mean ± SD of 20 volunteers. Specific differences were examined by an analysis of variance (ANOVA) and a multiple range test (Duncan’s test). The data were processed using the SPSS 21.0 software package (SPSS Inc., Chicago, IL, USA) and the level of significance was set at *p* < 0.05.

## 4. Conclusions

The results show an extensive degradation of Cy and Dp glycosides from maqui as they pass through the digestion system with a great variety of metabolites, including their phase II conjugates. However, none of the parental anthocyanins were found.

The results place sucralose as the sweetener which provided the greatest bioavailability for most metabolites. It significantly surpassed the bioavailability provided by stevia and sucrose. However, there are some cases where stevia also had a significantly higher bioavailability than sucralose and sucrose. Nevertheless, sucrose does not provide a higher bioavailability, nor sucralose or stevia, in any of the cases.

Considering the differences of bioavailability between sweeteners, this study proposes two non-caloric sweetener alternatives (sucralose and stevia) in order to reduce the consumption of sucrose, a high calorie sweetener that is directly related with some metabolic diseases such as type 2 diabetes and obesity. This research study contributes new information for the beverage industry, by providing evidence that supports the development of alternative juices, sources of bioactive compounds, using diverse sweeteners that more adequately fit with the current market demands. On the other hand, the different bioavailability described in regard to widely-recognized bioactive compounds would allow further exploring the current interest in them for the prevention of health disturbances with additional nutritional trials.

## Figures and Tables

**Table 1:** Anthocyanins composition of the maqui-citrus juices.

Beverages	Anthocyanins ^Z^ (mg/100 mL)							
	Dp 3-*O*-sam-5-*O*-glc	Dp 3,5-*O*-diglc	Cy 3 *O*-sam-5-*O*-glc + Cy 3,5-*O*-diglc	Dp 3-*O*-sam	Dp 3-*O*-glc	Cy 3-*O*-sam	Cy 3-*O*-glc	TOTAL
Stevia	3.06 ± 0.12 ^Y^	3.59 ± 0.02	1.54 ± 0.02	1.09 ± 0.01	2.87 ± 0.02	0.40 ± 0.01	0.54 ± 0.01	13.1 ± 0.2
Sucralose	3.19 ± 0.05	3.51 ± 0.01	1.51 ± 0.01	1.11 ± 0.01	3.02 ± 0.01	0.41 ± 0.01	0.57 ± 0.01	13.3 ± 0.1
Sucrose	3.19 ± 0.01	3.36 ± 0.09	1.38 ± 0.01	1.09 ± 0.01	2.90 ± 0.01	0.40 ± 0.01	0.55 ± 0.01	12.9 ± 0.2
*p*-value	>0.05 ^N.s.^	>0.05 ^N.s.^	>0.05 ^N.s.^	>0.05 ^N.s.^	>0.05 ^N.s.^	>0.05 ^N.s.^	>0.05 ^N.s.^	>0.05 ^N.s.^

^Z^ Cy, cyanidin; Dp, delphinidin; Glc, glucoside; Sam, sambubioside. ^Y^ Concentration of anthocyanins in samples presented as means ± SD (*n* = 3). The quantification of anthocyanins was done on UV chromatograms recorded at 520 nm as cyanidin-3-*O*-glucoside at 520 nm. N.s., not significant.

**Table 2:** Qualitative analysis of phenolic acids and anthocyanin metabolites detected in urine samples in non-hydrolyzed urine after the ingestion of the juices developed in the present work.

Compound	RT (min)	Precursor Ion	Product Ion	Fragmentation (V)	CE (V)	Polarity
*Cyanidin metabolites*						
Cyanidin (Cy)	8.81	287.0	137.0	100	20	Positive
Cy glucoside	N.f.	449.0	287.0	100	20	Positive
Cy diglucoside	N.f.	743.0	287.0	100	20	Positive
Cy sambubioside	N.f.	581.0	287.0	100	20	Positive
CYA sambubioside-glucoside	N.f.	611.0	287.0	100	20	Positive
*Delphinidin metabolites*						
Delphinidin (Dp)	5.18	303.0	229.0/257.0	100	20	Positive
Dp glucoside	N.f.	465.0	303.0	100	20	Positive
Dp diglucoside	N.f.	627.0	303.0	100	20	Positive
Dp sambubioside	N.f.	597.0	303.0	100	20	Positive
Dp sambubioside-glucoside	N.f.	759.0	303.0	100	20	Positive
*Caffeic acid metabolites*						
Caffeic acid (CA)	3.25	179.1	135.0	70	15	Negative
CA Glucuronide	2.40	355.1	179.1	70	15	Negative
CA diglucuronide	N.f.	531.1	179.1	70	15	Negative
CA Sulfate	2.99	259.1	179.1	70	15	Negative
CA Glucuronide-Sulfate	1.95	435.1	179.1	70	15	Negative
CA disulfate	N.f.	339.1	179.1	70	15	Negative
*Catechol metabolites*						
Catechol (CAT)	5.04	109.0	67.0	80	6	Negative
CAT Glucuronide	N.f.	286.0	109.0	80	6	Negative
CAT diglucuronide	2.83	461.0	109.0	80	6	Negative
CAT Sulfate	1.59	189.0	109.0	80	6	Negative
CAT Glucuronide-Sulfate	1.38	365.0	109.0	80	6	Negative
CAT disulfate	N.f.	269.0	109.0	80	6	Negative
*3,4*-*Dihidroxifenilacetic acid metabolites*						
3,4-Dihidroxifenilacetic acid (DHPAA)	1.80	166.8	123.2	70	5	Negative
DHPAA Glucuronide	1.58	342.8	166.8	70	5	Negative
DHPAA diglucuronide	1.04	518.8	166.8	70	5	Negative
DHPAA Sulfate	1.14	246.8	166.8	70	5	Negative
DHPAA Glucuronide-Sulfate	0.74	422.8	166.8	70	5	Negative
DHPAA disulfate	1.07	326.8	166.8	70	5	Negative
*Hippuric acid metabolites*						
Hippuric acid (HA)	2.55	178.0	134.4	80	5	Negative
HA Glucuronide	1.70	354.0	178.0	80	5	Negative
HA diglucuronide	0.59	530.0	178.0	80	5	Negative
HA Sulfate	1.78	258.0	178.0	80	5	Negative
HA Glucuronide-Sulfate	N.f.	434.0	178.0	80	5	Negative
HA disulfate	N.f.	338.0	178.0	80	5	Negative
*Gallic acid metabolites*						
Gallic acid (GA)	0.71	169.0	125.0	70	10	Negative
GA Glucuronide	N.f.	345.0	169.0	70	10	Negative
GA diglucuronide	N.f.	521.0	169.0	70	10	Negative
GA Sulfate	N.f.	249.0	169.0	70	10	Negative
GA Glucuronide-Sulfate	N.f.	425.0	169.0	70	10	Negative
GA disulfate	N.f.	329.0	169.0	70	10	Negative
*Trans.*-*ferulic acid metabolites*						
*Trans.*-ferulic acid (TFA)	4.46	192.8	133.8	20	5	Negative
TFA Glucuronide	4.25	368.8	192.8	20	5	Negative
TFA diglucuronide	1.74	544.8	192.8	20	5	Negative
TFA Sulfate	3.56	272.8	192.8	20	5	Negative
TFA Glucuronide-Sulfate	N.f.	448.8	192.8	20	5	Negative
TFA disulfate	1.32	352.8	192.8	20	5	Negative
*2,4,6*-*Trihidrobenzaldehid metabolites*						
2,4,6-Trihidrobenzaldehid (THBA)	5.10	153.1	106.8	90	18	Negative
THBA Glucuronide	5.08	329.1	153.1	90	18	Negative
THBA diglucuronide	N.f.	505.1	153.1	90	18	Negative
THBA Sulfate	1.46	233.1	153.1	90	18	Negative
THBA Glucuronide-Sulfate	N.f.	409.1	153.1	90	18	Negative
THBA disulfate	N.f.	313.1	153.1	90	18	Negative
*Trans.*-*isoferulic acid metabolites*						
*Trans.*-isoferulic acid (TIFA)	1.46	193.7	134.7	70	5	Negative
TIFA Glucuronide	N.f.	366.7	193.7	70	5	Negative
TIFA diglucuronide	N.f.	545.7	193.7	70	5	Negative
TIFA Sulfate	1.45	273.7	193.7	70	5	Negative
TIFA Glucuronide-Sulfate	N.f.	449.7	193.7	70	5	Negative
TIFA disulfate	N.f.	353.7	193.7	70	5	Negative
*Vanillic acid metabolites*						
Vanillic acid (VA)	3.18	167.0	151.8	100	15	Negative
VA Glucuronide	1.57	343.0	167.0	100	15	Negative
VA diglucuronide	N.f.	519.0	167.0	100	15	Negative
VA Sulfate	1.14	247.0	167.0	100	15	Negative
VA Glucuronide-Sulfate	0.93	423.0	167.0	100	15	Negative
VA disulfate	1.13	327.0	167.0	100	15	Negative

N.f., not found.

## Abbreviations

The following abbreviations are used in this manuscript:

ANOVA	Analysis of Variance
CA	Caffeic Acid
CAT	Catechol
CEIC	Ethical Committee of Clinical Studies
Cy	Cyanidin
Dp	Delphinidin
DHPAA	3,4-Dihidroxifenilacetic Acid
GA	Gallic Acid
Glc	Glucoside
Gln	glucuronide
HA	Hippuric Acid
LDL	Low Density Lipoprotein
MRM	Multiple Reaction Monitoring
RP-HPLC-DAD	Reverse-Phase High Performance Liquid Chromatography Diode Array Detector
Sam	Sambubioside
S	sulfate
TFA	*Trans.*-Ferulic Acid
THBA	2,4,6-Trihidrobenzaldehid
TIFA	*Trans.*-IsoFerulic Acid
UHPLC-ESI-MS/MS	Ultra-High Performance Liquid Chromatography ElectroSpray Ionization Mass Spectrometry
VA	Vanillic Acid
WHO	World Health Organization
